# Exploring the utilities of rice straw black liquor (part XVI): nano (zinc/lignin) hybrid for safe polyurethane films with enhanced antimicrobial, mechanical, and UV-protecting properties

**DOI:** 10.1038/s41598-026-48917-1

**Published:** 2026-04-30

**Authors:** Galal A. M. Nawwar, Ahmed M. Youssef, Hoda Sabry Othman

**Affiliations:** 1 Green Chemistry Department, National Research Centre, 33 El-Bohouth St. (former El-Tahrir St.), Dokki, 12622 Giza Egypt; 2 Packaging Materials Department, National Research Centre, 33 El-Bohouth St. (former El-Tahrir St.), Dokki, 12622 Giza Egypt; 3 Center for Converging Sciences and Emerging Technology (CoSET), Benha National University (BNU), Al Obour, 13518 Egypt

**Keywords:** Polyurethane, Food packaging, Sustainablity, Zinc lignin hybrid, Chemistry, Environmental sciences, Materials science, Nanoscience and technology

## Abstract

Green composites are increasingly attracting interest due to their potential to address environmental issues by merging sustainable fillers with biodegradable polymers. The current study investigates the incorporation of zinc lignin hybrid (Zn-LSF) nanoparticles into a polyurethane matrix at varying ratios. The hybrid’s elemental composition was determined using X-ray fluorescence (XRF), and Energy-dispersive X-ray spectroscopy **(**EDAX). The functional groups of both the hybrid and the prepared polyurethane films were identified by Fourier-transform infrared spectroscopy (FTIR). Scanning electron microscopy (SEM) was employed to analyze the morphology of the Zn-LSF/polyurethane films. Mechanical properties and permeability were also evaluated. Furthermore, the antimicrobial activity and toxicity of the composites were assessed. The results demonstrated that the zinc lignin hybrid is non-toxic, provides antimicrobial properties, and enhances the mechanical strength of polyurethane along with a UV-shielding effect for the prepared polyurethane films. These findings suggest that, the prepared composites have potential as a sustainable, multifunctional additive for safe polyurethane food packaging.

## Introduction

Fresh products, like fruits and vegetables, tend to spoil quickly as they are subjected to contamination by bacteria, yeast, and/or molds from the external environment^1^. Also, most polymeric films used in modified atmosphere packaging have insufficient water vapor permeability, causing moisture buildup from fresh products^2,3^. This leads to high humidity and condensation within the package, fostering microbial growth and product spoilage. Temperature variations during storage and transport exacerbate this problem^4^, a situation that has intensified the demand for effective packaging protection.

In the same context, foods are exposed to light throughout the food supply chain, from harvest to consumption. This exposure, particularly to UV light, generates free radicals and reactive oxygen species that degrade essential food components like proteins, lipids, and vitamins, impacting nutritional value and shelf life. Consequently, UV-shielding packaging films are indispensable for safeguarding food products, enhancing food safety, extending shelf life, and reducing food waste^5,6^. A current approach to improving both UV resistance and antimicrobial activity in packaging films involves incorporating nano-UV shielding agents^7,8^. This strategy is based on the superior properties of nanomaterials, such as improved mechanical performance and antimicrobial activity^9^. Hybrid UV shielding additives, when incorporated into polymeric films, offer multi-functional benefits, particularly in polyurethane films.

Lignin, a prevalent amorphous biopolymer, is a key component due to its inherent UV-blocking, antibacterial, and antioxidant properties^10–12^. These properties arise from its diverse functional groups, including carboxylic, carbonyl, methoxyl, aliphatic, and phenolic groups, along with other chromophores. Specifically, lignin’s phenolic groups play a crucial role^13^. Their free radical scavenging ability not only enhances the thermal and oxidative stability of polymers but also contributes to lignin antioxidant and antimicrobial properties^14,15^. According to Feldman and Banu^16^, the combination of methoxy and phenolic hydroxyl groups in lignin forms hindered phenol structures, which can effectively capture free radicals. This ability to stabilize reactions against oxygen and its radical species is further supported by Ye et al.^17^, who demonstrated that lignin’s antioxidant activity increases proportionally with its phenolic hydroxyl content.

Lignin’s capacity to engage in both chemical and physical interactions with metal ions enables the creation of novel materials exhibiting improved characteristics. Hybrid materials composed of lignin and inorganic precursors demonstrate superior physicochemical properties compared to their pure counterparts^18^. Within our scientific program, we are dedicated to exploring the potential of biomass, particularly rice straw^19–27^. We have developed novel lignin hybrids that combine silica and fatty acids^18,28–32^. Silica is a versatile material with diverse applications^33,34^. Due to the presence of surface hydroxyl groups, silica exhibits hydrophilicity, making it compatible with polymeric material through chemical interactions between the polymer matrix and silica’s silanol groups (Si-OH), improving the mechanical properties of polymeric films alongside enhancing antimicrobial activity, UV protection, and scratch resistance^18,35^. In addition, studies have shown that the lipids and fatty acids extracted from rice straw can be converted into bioactive salts that enhance the performance of numerous industrial products^36–38^. Developing such lignin/silica/fatty acid hybrids from lignocellulosic biomass, driven by potential cost savings, is gaining significant attention^28–32,39–41^.

Polymer materials are increasingly in demand for food packaging^42^, making it a major growth market. While conventional, petroleum-based plastics have revolutionized packaging, their non-degradability has led to severe environmental issues, including threats to aquatic life and air quality degradation. The rise of food industries has exacerbated this problem, prompting the development of biodegradable polymers as a sustainable alternative^43^. The ASTM defines biodegradable polymers as materials degraded by naturally occurring microorganisms^44,45^. Derived from renewable resources, these polymers offer properties comparable to the conventional ones like PET, PP, and PE. Their degradation results in environmentally friendly products, including water, CO_2_, inorganic compounds, or biomass, preventing waste accumulation and benefiting the environment.

The global manufacturing capacity for bioplastics, such as polyurethane, polylactic acid (PLA), and polybutylene adipate-co-terephthalate (PBAT), is expected to surge from 2.42 million tonnes in 2021 to 7.6 million tonnes by 2026, as reported by European Bioplastics and Nova-Institute (2021)^46^.

**Fig. 1:**
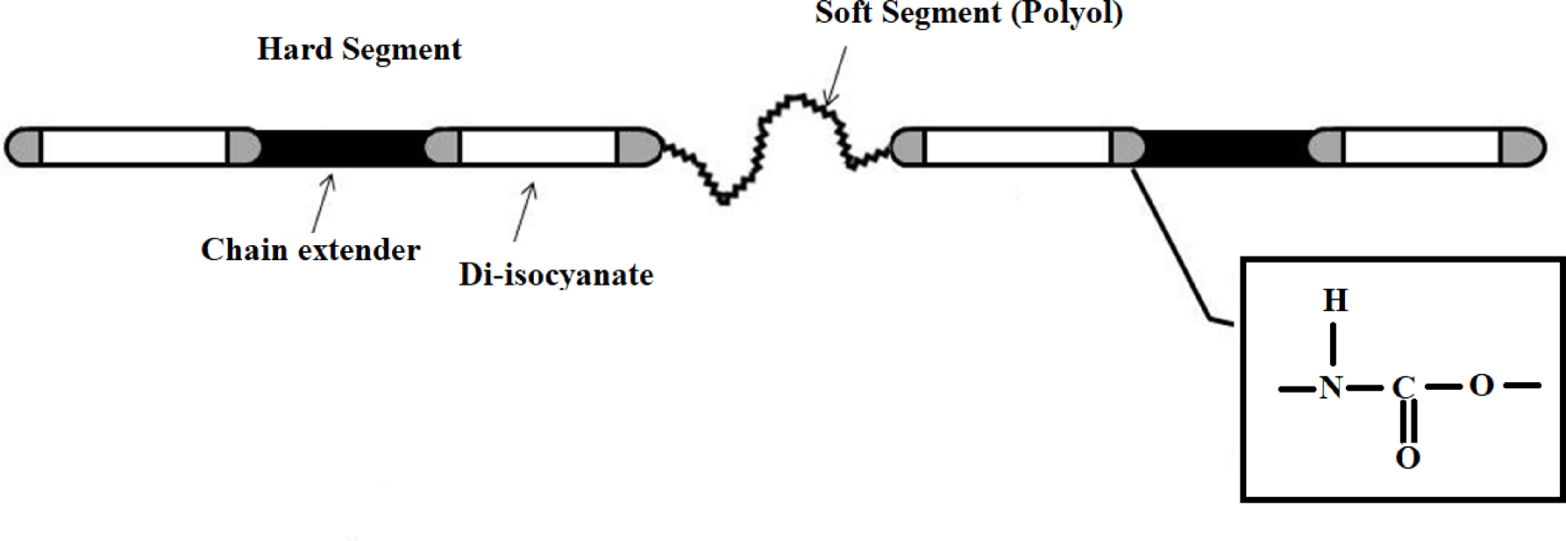
Polyurethane structure.

Polyurethane, a unique polymeric material with a wide range of physical and chemical properties, is a notable polymer for fresh produce packaging due to its controllable gas permeability with respect to temperature^47^. Polyurethane is a segmented polymer with alternating hard segments and soft segments (Fig. 1)^48^. Such structure provides polyurethane with a broad spectrum of excellent mechanical, physical, and chemical properties, from flexible coatings to robust construction materials, along with exceptional biocompatibility, enabling extensive customization for manufacturers. It has been extensively tailored to meet the highly diversified demands of modern technologies such as coatings, adhesives, fiber, foams, and thermoplastic elastomers^48–50^. The biodegradability of polyurethanes is determined by the chemical composition of their segments, particularly the soft segment. Choosing a polyester polyol results in readily biodegradable polyurethanes, whereas polyether-based polyurethanes exhibit resistance to biodegradation^3,48^.

Antimicrobial polyurethane films are of significant interest in this regard. Enhancing these films with antimicrobial compounds within the polymer matrix is a key strategy to prevent microbial colonization and food spoilage^51,52^. However, selecting the appropriate antimicrobial agent is critical, as the agent’s properties can directly influence the final product’s characteristics and functionality.

Our previous work successfully synthesized lignin/silica hybrid metal complexes that, when incorporated into rubber matrices, significantly improved their rheological characteristics, thermal stability, electrical conductivity, and mechanical and antimicrobial properties^28–32^. These complexes also served as effective multifunctional nano-pigments, producing colored coatings with excellent UV resistance and antimicrobial activity^18^.

Recognizing the promising properties of the Zn-Lignin/Silica/fatty acid hybrid^31^, we chose to integrate it into polyurethane to develop multifunctional biodegradable films. These films, prepared with nano-additive concentrations of 1%, 5%, and 10% (by mass), are anticipated to hold significant potential for multi-functional enhancement, demonstrating UV resistance, antioxidant, and antimicrobial activities.

## Materials and methods

### Materials

Thermoplastic polyurethane (PU) elastomer with a specific gravity of 1.20 g/cm^3^ was purchased in pellet form from Bayer, Germany. The Normal Human Skin Fibroblast (HSF) cell line was purchased from the American Type Culture Collection (ATCC), Virginia, (USA). DMEM Cell culture medium was obtained from Invitrogen-Life Technologies. MTT was purchased from Acros Organics™.

### Synthesis of zinc/Lignin hybrid (Zn-LSF)

Zn-LSF hybrid was synthesized from rice straw pulping black liquor, following our previously published method^31^.

### Preparation of polyurethane/Zn-LSF bionanocomposite films

PU/Zn-LSF bionanocomposite films were prepared by melt-blending PU with varying ratios of Zn-LSF nanoparticles (Table 1) using a Brabender PlastiCorder at 180 °C for 10 min (60 rpm). Pure PU served as a control. The resulting mixtures were then compression-molded at 180 °C and 150 kg/cm² for 3 min using a hydraulic press. The mold, consisting of a steel spacer between Teflon sheets and steel slabs, was cooled under load to 50–60 °C. Samples were labeled according to nanoparticle concentration (Table 1).

**Table 1:** PU/Zn-LSF bionanocomposite film formulations: composition, compounding protocol, and sample labeling.

Composite code	Constituents (wt%)		Compounding conditions		
	Matrix	Nanofiller	Temperature [°C]	Roller speed [rpm]	Mixing time [min]*
	TPU	Zn-LSF, %			
Pure PU	100	-	180	70	10
PU/Zn-LSF-1%	99	1	180	70	10
PU/Zn-LSF- 5%	95	5	180	70	10
PU/Zn-LSF-10%	90	10	180	70	10

### Characterizations

#### X-ray fluorescence (XRF) spectrometer

The elemental composition of the Zn-LSF hybrid was determined using wavelength dispersive X-ray fluorescence spectrometry (WDXRF) with a PANalytical Axios advanced sequential spectrometer (2005).

#### Scan electron microscope and energy-dispersive X-ray spectroscopy (EDAX)

The surface structure of the prepared samples was examined using scanning electron microscopy (SEM), JEOL JSM 6360LV (operating at 10–15 kV), attached to the EDAX unit. To enhance conductivity and prevent damage, samples were coated with a thin gold layer via sputter-coating before SEM analysis, with careful control of deposition rate and target distance.

#### Fourier-transform infrared (FT-IR) spectroscopy

FT-IR spectral analysis was conducted using a JASCO FT/IR-4100 LE spectrometer (Easton, MD, USA), operating in absorption mode, across a wavenumber range of 4000 –400 cm⁻¹.

#### Mechanical properties

The mechanical properties of the prepared biocomposites, specifically tensile strength (MPa) and elongation (%), were measured using an INSTRON 34SC-5 universal testing machine (USA) with a 5-N load cell and a constant crosshead speed of 2.5 cm/min. All tests were performed in triplicate, and the results are presented as mean values with standard deviations.

#### Ultraviolet resistance

The UV-resistance-resistance was measured according to ASTM D4587–91, where the films were exposed to a short- and long-wave UV lamp (4 watts, 245/312 nm wavelength) in a chamber at room temperature for 100 h.

#### Permeability assessments

The water vapor permeability was evaluated by using a Water Vapor Permeability Analyzer (GBI W303 (B), China), which was used to assess the amount of water vapor transmission rate (WVTR) *via* the cup method. Furthermore, WVTR was measured as the amount of water vapor transmitted over a unit area in a unit time under a precise temperature (38 °C) and humidity (4%) using the following standards (ASTM E96).

#### Antimicrobial activity of the prepared bionanocomposites

The antimicrobial activity of the prepared films was evaluated against *Staphylococcus aureus* (ATCC 6538), *Escherichia coli* (ATCC 8739), and *Candida albicans*. Overnight broth cultures of these microorganisms, grown at 37 °C for bacteria and 25 °C for fungi^53^, were used to prepare cell suspensions adjusted to a 0.5 McFarland standard (approximately 1 × 10⁵ CFU/mL). 150 µL of each suspension was inoculated into 25 mL of sterile buffered peptone water medium^54^. Samples were then introduced into these flasks, and the reduction in microbial counts was determined after 6 h of incubation using a shake flask method^55^. Bacterial counts were determined on nutrient agar after 24 h of incubation at 37 °C, while fungal counts were determined on potato dextrose agar after 36 h of incubation at 25 °C. Results are expressed as colony-forming units per milliliter (CFU/mL).

#### Cytotoxic effect on human cell lines

Mitochondrial-dependent cell viability was assessed by the reduction of yellow MTT (3-(4,5-dimethylthiazol-2-yl)-2,5-diphenyl tetrazolium bromide) to purple formazan.

All the following procedures were done in a sterile area using a Laminar flow biosafety cabinet Class II A2 (Manufactured by: Labconco). Cells were suspended in Dulbecco’s Modified Eagle medium (DMEM), 1% antibiotic-antimycotic mixture (10,000U/ml Potassium Penicillin, 10,000 µg/ml Streptomycin Sulfate and 25 µg/ml Amphotericin B) and 1% L-glutamine and 5% fetal bovine serum at 37 °C under 5% CO_2_ using CO_2_ incubator ( Sartorius stedium).

Cells were batch cultured for 10 days, then seeded at concentration of 10 × 10^3^ cells/well in fresh complete growth medium in 96-well plastic plates at 37 °C for 24 h under 5% CO_2_ either alone (negative control) or with different concentrations of Zn-LSF hybrid to give a final concentration of (2000, 1000, 500, 250, 125, 62.5, 31.25, 15.625 ug/ml). After 48 h of incubation, medium was aspirated, 20 ul MTT salt (2.5 µg/ml) were added to each well and incubated for further four hours at 37 °C under 5% CO_2_. To stop the reaction and dissolving the formed crystals, 200µL of 10% Sodium dodecyl Sulphate (SDS) in 0.01 M HCL was added to each well and incubated overnight at 37 °C^56,57^. The absorbance was then measured using a microplate multi-well reader (Bio-Rad Laboratories Inc., model 3350, Hercules, California, USA) at 595 nm and a reference wavelength of 620 nm.

Viability = absorbance of drug / absorbance of control x 100.

Cytotoxicity = 100- viability.

## Result and discussion

### X-ray fluorescence (XRF) analysis

The high loss on ignition (LOI 58.8%) and zinc oxide shown in Table 2 strongly indicate that zinc lignate and zinc salts of saponified fatty acids, formed during alkaline pulping of rice straw, are the major constituents accompanied by a minor presence of zinc silicate.

**Table 2:** XRF of Zn- LSF Hybrid.

Main constituents	(wt%)
SiO_2_	1.63
Al_2_O_3_	0.08
Fe_2_O_3_	0.01
MgO	0.08
CaO	0.04
ZnO	35.45
Na_2_O	1.65
K_2_O	0.22
P_2_O_5_	0.02
SO_3_	0.05
Cl	1.96
LOI	58.8
NiO	0.004
CuO	0.006

### Morphology of the prepared PU/Zn-LSF films

**Fig. 2:**
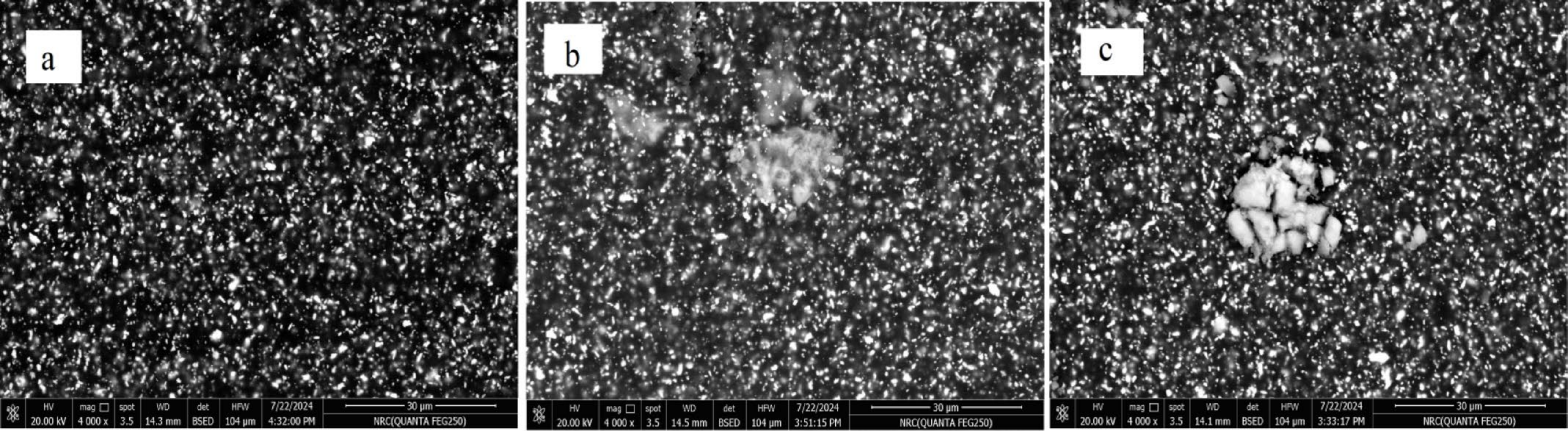
SEM images of PU/Zn-LSF films: (**a**) Pure TPU, (**b**) 1%, (**c**) 10% Zn-LSF nanoparticles.

The morphology of the PU/Zn-LSF nanocomposite films, as influenced by nanoparticle loading, was examined via SEM (Fig. 2). While the pristine polyurethane exhibited a characteristically homogeneous surface (Fig. 2a), the addition of Zn-LSF (1–10%) induced noticeable structural changes. As the nanoparticle concentration increased, the film surfaces became progressively rougher and less uniform (Fig. 2b, c). This transition was marked by the formation of micro-channels, voids, and localized aggregation, all of which intensified in direct correlation with the nanoparticles concentration in the polymer matrix.

**Fig. 3:**
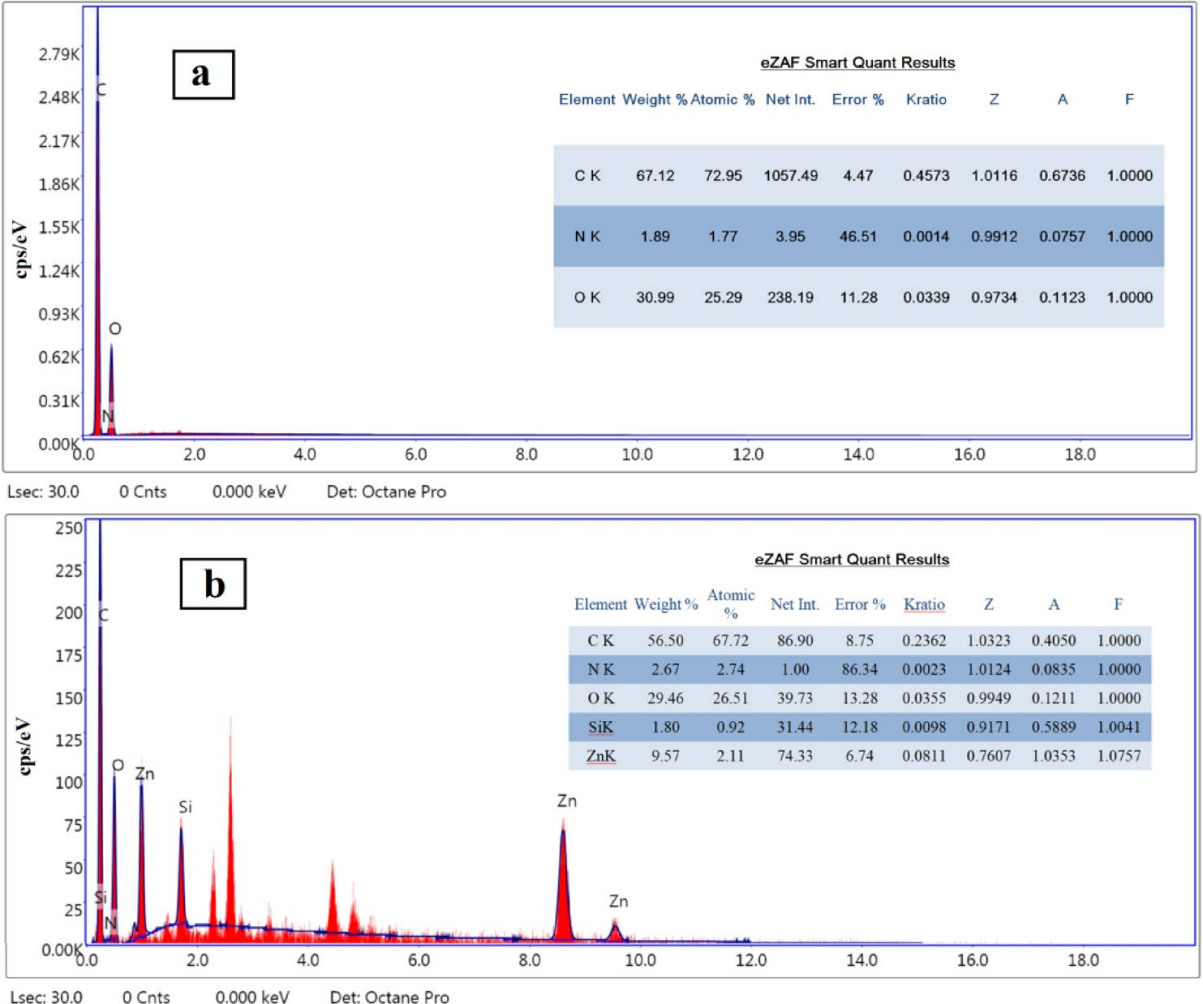
EDAX analysis of the nanocomposites film: (**a**) pure PU film and (**b**) PU/Zn-LSF nanocomposites film containing 10% Zn-LSF nanoparticles.

Furthermore, EDAX of the prepared PU/Zn-LSF composite film (Fig. 3b) provides a graphical representation of silica, zinc and nitrogen percentages, confirming the incorporation of the Zn-LSF complex into the polyurethane matrix.

### FTIR Spectra

**Fig. 4:**
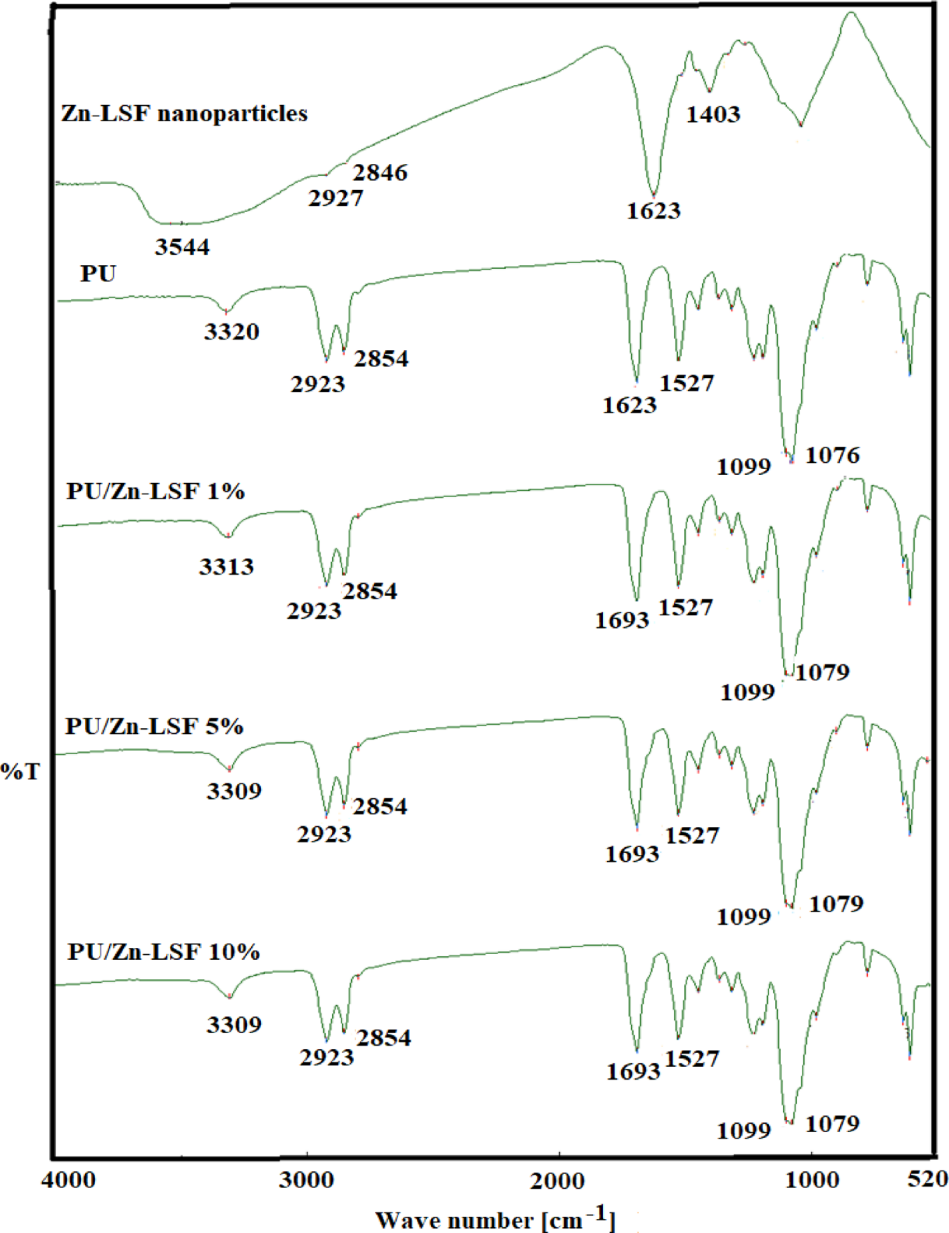
FTIR Spectra of Zn-LSF nanoparticles, Polyurethane film, and PU films loaded with 1%, 5%, 10% Zn-LSF.

The FT-IR spectrum of the prepared lignin hybrid (Zn-LSF) (Fig. 4) exhibited characteristic absorption bands. A broad peak at 3544–3428 cm⁻¹ corresponds to the stretching vibrations of alcoholic and phenolic OH groups in lignin and NH stretching of polyurethane^58,59^; while a peak at 2927 cm⁻¹ indicates alkyl groups. The sharp band at 1623 cm⁻¹ is attributed to carbonyl stretching in fatty acids^60^, and the band at 1403 cm⁻¹ signifies C–O stretching vibrations. In the Zn (LSF)-polyurethane spectra, new shoulders appeared at 1079 cm⁻¹ and 1693 cm⁻¹, indicating the incorporation of the lignin hybrid and polyurethane CO, respectively^30,59^.The intensity of these shoulders increased with increasing Zn-LSF concentration in the polyurethane matrix. It is worthy of mention that slight shifts were observed in the IR spectra of the prepared films after UV exposure.

### Mechanical properties of the prepared PU/Zn-LSF bionanocomposites

**Fig. 5:**
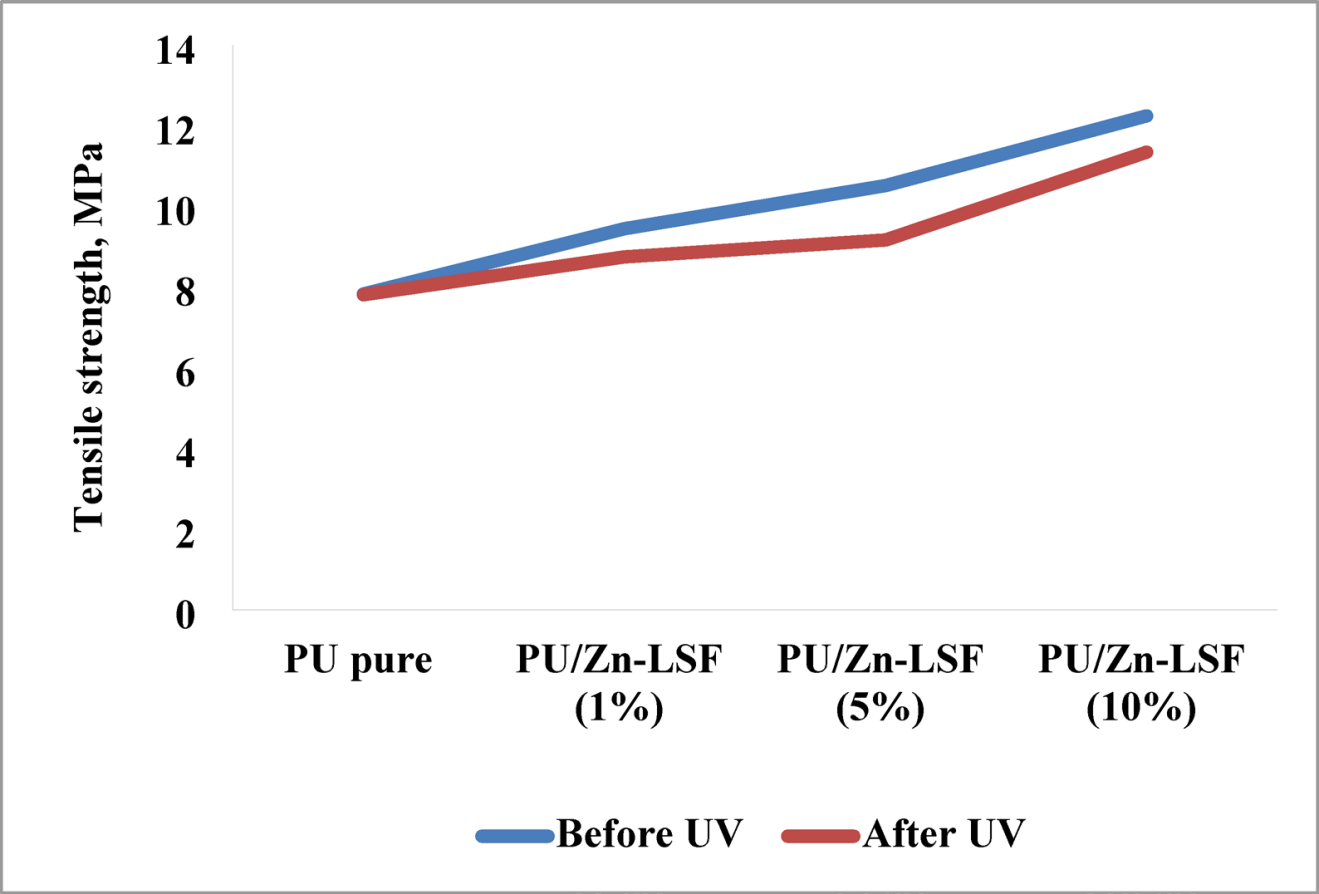
Effect of Zn-LSF concentration on Tensile strength of the prepared PU films.

**Fig. 6:**
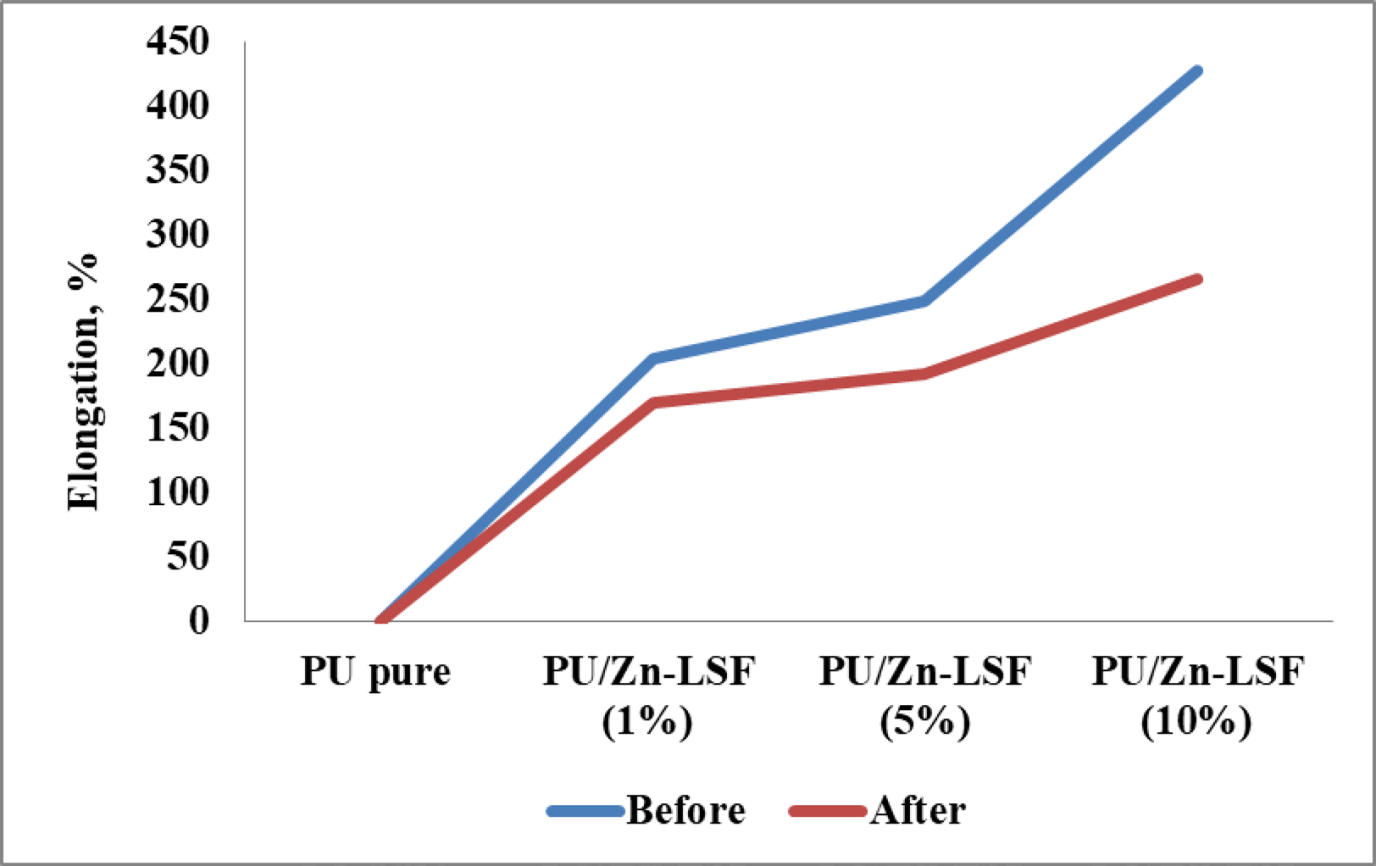
Effect of Zn-LSF concentration on elongation of the prepared PU films.

The mechanical properties of polyurethane (PU) composites reinforced with Zn-LSF nanoparticles were investigated, both before and after UV exposure. Table 3 reveals that incorporating Zn-LSF significantly affected the PU’s tensile strength (TS) (Fig. 5), and elongation (Fig. 6). Pure PU (0% Zn-LSF) showed a TS of 7.83 MPa and an elongation of 204.28% prior to UV treatment, with minimal changes post-treatment. However, adding just 1% Zn-LSF increased the TS to 9.45 MPa and elongation to 248.94% before UV exposure, demonstrating that even a small addition of Zn-LSF enhances the mechanical performance of the PU nanocomposites. However, both values slightly decreased after UV exposure (8.75 MPa and 192.71%). By increasing Zn-LSF loading to 5% in the polyurethane matrix, further enhanced mechanical properties were achieved, with tensile strength reaching 10.52 MPa and elongation significantly increasing to 428.23% before UV treatment. These increased values on UV exposure slightly reduced to 9.17 MPa and 266.08%. The same changes occurred at 10% Zn-LSF loading, where tensile strength peaked at 12.25 MPa (before UV) and reduced to 11.35 MPa (after UV). Also, elongation decreased to 302.16% after UV treatment, from 456.80% before. Overall, the obtained data indicates that Zn-LSF can act as an effective reinforcing agent, with greater impact at higher loadings; this linearity was also shown on UV exposure.

**Table 3:** Mechanical characteristics of the prepared PU/Zn-LSF bionanocomposites.

Sample	Tensile strength, MPa		Elongation, %	
	Before UV	After UV	Before UV	After UV
PU pure	7.83	7.81	204.28	170.22
PU/Zn-LSF (1%)	9.45	8.75	248.94	192.71
PU/Zn-LSF (5%)	10.52	9.17	428.23	266.08
PU/Zn-LSF (10%)	12.25	11.35	456.80	302.16

### Permeability assessments of the fabricated PU/Zn-LSF bionanocomposites

Table 4 presents the permeability of PU/Zn-LSF bionanocomposites, showing the impact of Zn-LSF nanoparticles on the barrier properties of polyurethane films. The oxygen transmission rate (OTR) and water vapor transmission rate (WVTR) were evaluated. Pure polyurethane exhibited an OTR of 9.65 (cc/m²·day) and a WVTR of 78.65 g/(m²·day), indicating moderate oxygen barrier and high water vapor permeability. Adding 1% Zn-LSF slightly increased OTR to 10.38 (cc/m²·day), suggesting a modest improvement in oxygen permeability, but decreased WVTR to 60.41 g/(m²·day), indicating that even a small nanoparticle presence begins to create a more tortuous path for water vapor molecules, thus improving the moisture barrier.

At 5% Zn-LSF, OTR significantly increased to 24.30 (cc/m²·day), possibly due to increased micro-channels or voids within the matrix that facilitate oxygen transmission, while WVTR dropped to 40.13 g/(m²·day), enhancing the water vapor barrier. With 10% Zn-LSF, OTR slightly increased to 25.82 (cc/m²·day), and WVTR further decreased to 31.92 g/(m²·day), demonstrating a substantial enhancement in water vapor barrier. Thus, Zn-LSF creates a trade-off: increased loadings reduce water vapor permeability (lower WVTR) but increase oxygen permeability (higher OTR).

The observed dual effect suggests that Zn-LSF particles modify the PU matrix’s microstructure. This likely involves introducing micro-voids, enhancing oxygen transmission, alongside the creation of tortuous pathways, impeding water vapor movement. Such insights are crucial for tailoring the composite’s properties for applications requiring specific moisture or oxygen barrier characteristics.

**Table 4:** The permeability of the produced PU/Zn-LSF bionanocomposites.

Films name	OTR, cc/(m^2^.day)	WVTR, g/(m^2^.day)
PU pure	9.65	78.65
PU/Zn-LSF (1%)	10.38	60.41
PU/Zn-LSF (5%)	24.30	40.13
PU/Zn-LSF (10%)	25.82	31.92

### Antimicrobial activity

The antimicrobial activity of synthesized UV-resistant nano Zn-LSF hybrid films is presented in (Table 5). The hybrid significantly reduced bacterial and fungal counts: *Staphylococcus aureus* by 81.80%, *Escherichia coli* by 83.81%, and *Candida albicans* by 83.05%, compared to the control. This antimicrobial activity could be attributed to the inherent antimicrobial properties of lignin and zinc soap components within the hybrid (cf. Table 1)^18^ and to the synergistic effect of zinc ions (Zn²⁺) with acid radicals. Notably, the effectiveness increased with higher hybrid concentrations. Furthermore, the nanosize of the synthesized Zn-LSF hybrid facilitates cellular entry, leading to reduced bacterial metabolic activity. The hydrophobic nature of these lignin-based nanoparticles also allows them to intercalate with bacterial membrane lipids, disrupting the envelope.

**Table 5:** Log reduction % for different coating films.

		C	B	Zn 1%	Zn 5%	Zn 10%
*S. aureus*	Bacterial counts	6.49	6.00	4.23	2.99	1.18
	Log reduction %		7.55	34.82	53.92	81.80
*E. coli*	Bacterial counts	6.18	5.17	3.22	2.30	1.00
	Log reduction %		16.66	47.89	62.78	83.81
*C. albicans*	Bacterial counts	5.90	5.00	3.50	2.10	1.00
	Log reduction %		15.25	40.67	64.40	83.05

### Cytotoxic effect on human cell lines

**Fig. 7:**
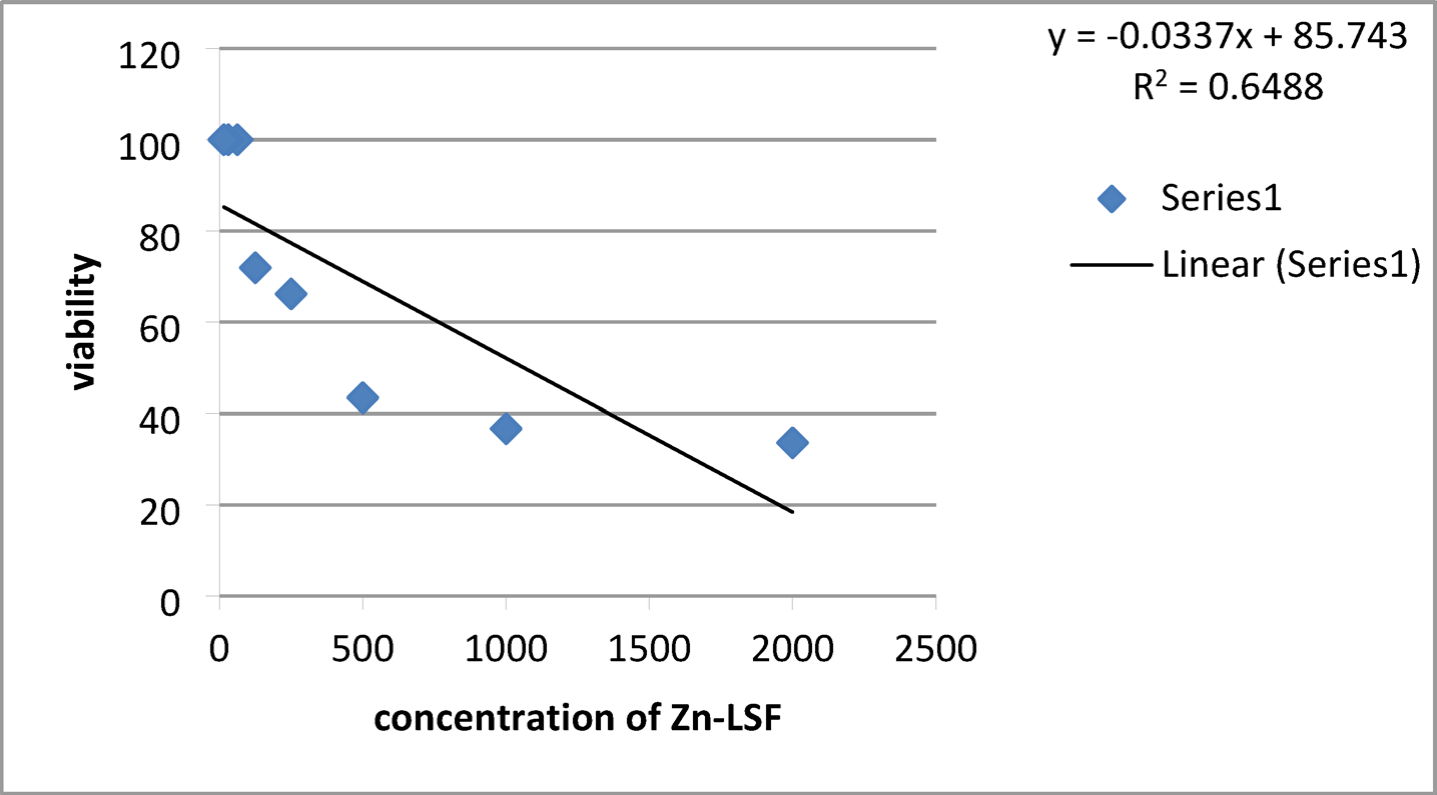
Effect of Zn-LSF hybrid on the viability of HSF cell line. IC_50_ : concentration (µg/mL) of the sample which causes the death of 50% of cells in 48 h.

The sample was tested against the normal human skin fibroblast (HSF) cell line. The Zn-LSF hybrid exhibited relatively low toxicity (Table 6; Fig. 7), with an IC_50_ value of 863.67 µg/mL and an IC_30_ of 478.29 µg/mL, indicating that a high concentration is required to induce cytotoxicity. Therefore, utilizing concentrations between 1% and 10% is both safe and effective. This range avoids the increased agglomeration (*Cf.* Figure 2) that typically triggers a decline in mechanical performance.

**Table 6:** Cytotoxicity of different concentrations of Zn-LSF hybrid.

Different dilution of Zn-LSF ( ug /ml)	Viability %	Cytotoxicity %
2000	33.6	66.4
1000	36.7	63.3
500	43.4	56.6
250	66.2	33.8
125	71.9	28.1
62.5	100	00
31.25	100	00
15.625	100	00

## Conclusion

The biomass-benign nano Zn-LSF hybrid, when incorporated into polyurethane, provided antimicrobial properties while also enhancing the mechanical properties of prepared films. These effects increased with an increase of the added concentration. While UV exposure generally improved the films’ mechanical properties in a concentration-dependent manner, the net gain was slightly lower than the initial increases observed prior to irradiation. A 5% concentration was selected as the Optimal Replacement Threshold (ORT) to balance non-toxicity, mechanical performance, and cost, while specifically avoiding the particle agglomeration seen at 10% loading. These results demonstrate the potential of rice straw-derived nano zinc lignin hybrid as a sustainable, multifunctional additive for biopolyurethane food packaging, especially for vegetables and fruits. The obtained PU-based biocomposites, offering both performance improvements and environmental and health safety, is the main finding in the presented work. The suggested optimum concentration is from 1 to 5% of the Zn hybrid. Further investigation is recommended.

## Data Availability

All data generated or analysed during this study are included in this published article.
